# Detection of milk powder in liquid whole milk using hydrolyzed peptide and intact protein mass spectral fingerprints coupled with data fusion technologies

**DOI:** 10.1002/fsn3.1430

**Published:** 2020-02-03

**Authors:** Lijuan Du, Weiying Lu, Yaqiong Zhang, Boyan Gao, Liangli Yu

**Affiliations:** ^1^ Department of Food Science and Technology School of Agriculture and Biology Institute of Food and Nutraceutical Science Shanghai Jiao Tong University Shanghai China; ^2^ China‐Canada Joint Lab of Food Nutrition and Health (Beijing) Beijing Technology & Business University (BTBU) Beijing China; ^3^ Department of Nutrition and Food Science University of Maryland College Park MD USA

**Keywords:** data fusion, intact protein fingerprints, milk adulteration, peptide fingerprints, principle component analysis

## Abstract

Detection of the presence of milk powder in liquid whole milk is challenging due to their similar chemical components. In this study, a sensitive and robust approach has been developed and tested for potential utilization in discriminating adulterated milk from liquid whole milk by analyzing the intact protein and hydrolyzed peptide using ultra‐performance liquid chromatography with quadrupole time‐of‐flight mass spectrometer (UPLC‐QTOF‐MS) fingerprints combined with data fusion. Two different datasets from intact protein and peptide fingerprints were fused to improve the discriminating ability of principle component analysis (PCA). Furthermore, the midlevel data fusion coupled with PCA could completely distinguish liquid whole milk from the milk. The limit of detection of milk powder in liquid whole milk was 0.5% (based on the total protein equivalence). These results suggested that fused data from intact protein and peptide fingerprints created greater synergic effect in detecting milk quality, and the combination of data fusion and PCA analysis could be used for the detection of adulterated milk.

## Graphical abstract

A sensitive and robust approach has been developed and tested for potential utilization in discriminating adulterated milk from liquid whole milk by analyzing the intact protein and hydrolyzed peptide using UPLC‐QTOF‐MS fingerprints combined with data fusion. The limit of detection of milk powder in liquid whole milk was 0.5% (based on the total protein equivalence). Results suggested that fused data from intact protein and peptide fingerprints created greater synergic effect in detecting milk quality, and the combination of data fusion and principle component analysis (PCA) analysis could be used for the detection of adulterated milk.

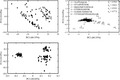

## 1 INTRODUCTION

Milk is the most important dairy source and food commodity worldwide. Milk adulteration is a global food safety and integrity issue due to its wide consumption. One of the widely known milk frauds is the illegal addition of nitrogen‐rich molecule such as melamine, which results in children hospitalized in China (Xin & Stone, 2008). Besides, the illegal addition of some low‐cost botanical proteins, such as soybean or pea protein, into milk might induce serious food allergies and threaten human health (Sanchez‐Monge et al., 2004; Satoh, Nakamura, Komatsu, Oshima, & Teshima, 2011; Yang, Zheng, Soyeurt, Yang, & Wang, 2018). Except for the adulterants mentioned above, milk powder is another type of liquid milk adulterant (Calvano, Monopoli, Loizzo, Faccia, & Zambonin, 2013). And this type of milk adulteration is more difficult to be detected, since liquid whole milk and adulterated milk have almost similar fundamental chemical properties. However, milk powder is a relative low‐cost milk ingredient, and thermal processing might have changed its nutritional values through chemical reactions such as the propagating chain and carbonylation reactions (Mauron, 1981; Singh & Creamer, 1991; Boekel, 1998). The adulteration with milk powder in liquid whole milk may encroach the consumers' economic rights, through it might not be pernicious to human health. Therefore, it is necessary to develop accurate and reliable analytical methods for adulterated milk detection.

Targeted analyses were the general strategies in detecting liquid whole milk adulteration in the past years (Cozzolino et al., 2001). These methods could characterize and quantify the targeted specific adulterant compounds using standards (Cozzolino et al., 2001). However, targeted detections are not suitable for detecting unknown adulterants, especially any unexpected illegal adulterants in milk such as melamine. For milk powder illegally added into the liquid whole milk, the conditions are even worse. Since the milk powder and liquid whole milk have almost similar chemical compositions, and no other chemical or biohazards were added, regular detection approaches are difficult to differentiate them. To detect milk powder adulterated to liquid milk more effectively, nontargeted techniques, including electronic nose (Yu, Wang, & Xu, 2007), ultraviolet, and visible spectroscopies (Madkour & Moussa, 1989), were utilized in previous studies. But these nontargeted detection methods still have limitations, such as low sensitivity, narrow selectivity, and depending on empirical parameters without appropriate statistical analysis. High sensitivity and reliability of nontargeted analytical approaches including data processing methods are urgently needed in detecting liquid whole milk adulterated with milk powder.

Mass spectrometry is one of the effective technologies in analyzing and identifying either intact protein or hydrolyzed peptide fragments of different milk proteins, including that in cow, goat, camel, yak, and buffalo milks (Du et al., 2018; Mamone, Picariello, Caira, Addeo, & Ferranti, 2009; Nardiello, Natale, Palermo, Quinto, & Centonze, 2018; Vincent, Elkins, Condina, Ezernieks, & Rochfort, 2016a; Vincent, Ezernieks, et al., 2016b; Yang et al., 2014). This study aims to investigate the application of LC‐MS technology in differentiating the presence of milk powder from liquid whole milk by analyzing the intact protein and peptide. The combination of analytical approaches and chemometrics might be helpful in obtaining a more complete and precise model to characterize liquid whole milk and adulterated powder adulterated milk.

Data fusion is a framework data processing approach, mainly to merge data from several different data sources (Ramos, Ruisánchez, & Andrikopoulos, 2008). Data fusion has been used in detecting food adulterations, such as in identifying botanical origin of Sicilian honeys, classifying different variety wine samples and grape juice (Cozzolino, Cynkar, Shah, & Smith, 2012; Márquez, López, Ruisánchez, & Pilar Callao, 2016; Ramos et al., 2008; Rosa, Leone, Scattareggia, & Chiofalo, 2018; Silvestri et al., 2014). Data fusion can be performed during different stages of chemometrics (Dearing, Thompson, Rechsteiner, & Marquardt, 2011), which have repercussions for fusion of discriminating results. As an alternative solution, data fusion could improve the classification ability of the models by using data from different sources, compare models between individual techniques (Gutiérrez et al., 2013; Vera et al., 2011). The merging of results from different measurements conducts a better description of the investigated results (Smilde, van der Werf, Bijlsma, van der Werff‐van der Vat, & Jellema, 2005). Therefore, this study was conducted to develop and validate a comprehensive model merging peptide and intact protein LC‐MS fingerprints data blocks. The results of this research suggested the potential of LC‐MS techniques in combination with common chemometric tools and data fusion in accurately and effectively differentiating the adulterated milk and liquid whole milk, thus protecting the consumer's interests.

## 2 MATERIALS AND METHODS

### 2.1 Materials and chemicals

Eighteen liquid milk samples were gifted by Nestle Corporation from Qingdao, Shandong, China. All the milk samples were stored at −20°C before analysis. The adulterants consist of four different brands of whole milk powders that were purchased from a local supermarket.

Bovine serum albumin (BSA) test kit was purchased from Beyotime Biotechnology (Shanghai, China); ammonium bicarbonate, dithiothreitol (DTT), iodoacetamide (IAA), and N‐tosyl‐L‐phenylalanine chloromethyl ketone (TPCK)‐modified trypsin were obtained from Sigma‐Aldrich. LC‐grade formic acid was obtained from ANPEL Laboratory Technologies Incorporated Company. Acetonitrile was of LC‐MS grade and purchased from Merck. Water was purified from distilled water using a Milli‐Q system (Millipore Laboratories).

### 2.2 Sample preparation

The frozen liquid whole milks were thawed at ambient temperature. One milk sample was randomly selected as the standard authentic sample, and all the adulterated milk samples were prepared using this sample. Ultra‐pure water was added to one aliquot of milk to produce a 10‐fold dilution; the diluted sample was centrifuged at 9,391 g for 20 min at 4°C, and the insoluble residue at the bottom of the solution and the film of fat at the top of the solution were removed. Protein solution was collected and diluted with ultra‐pure water to 0.5 mg/ml for analysis, with the concentration determined by BCA protein assay. This concentration of total protein content was then used to calculate the adulteration ratios of adulterated milk in liquid whole milk. Each milk sample was prepared in triplicate.

For adulterated milk, 20 mg of milk powder was weighed into a 15 ml polypropylene tube and dissolved in 10 ml water at 70°C. Milk powder solution (2 mg/ml) was centrifuged at 9,391 *g* for 20 min at 4°C, and the rest of the sample preparation was identical to that of liquid whole milk. All milk solutions were diluted to a protein concentration of 0.5 mg/ml according to BCA protein assay. Then, four reconstructed milk samples were mixed with liquid whole milk sample at 0.5%, 1%, 3%, 5%, and 10% based on the total protein equivalence measured by BCA protein assay. Three individual aliquots from one sample were separately prepared for all the samples. A total of 54 authentic milk samples and 60 adulterated milk samples were prepared and directly analyzed for intact proteins.

Extracted milk protein samples were processed to obtain the peptide hydrolyzates by a series of alkylation, acylation, and enzymatic reactions based on previous protocol (Lu, Liu, Gao, Lv, & Yu, 2017). Briefly, 1 ml of 100 mM ammonium bicarbonate was mixed with 1 ml of extracted milk protein samples. Then, 10 μl 1 M dithiothreitol (DTT) was added, and the mixture was incubated for 30 min at 50°C and followed by adding 30 μl of 1 M iodoacetamide (IAA). The mixture was incubated in dark at ambient temperature for 30 min. After that, 20 μl of 2 mg/ml trypsin solution was added into the mixture and the resulting solution was incubated for 12 hr at 37°C. The digestion was stopped by heating at 85°C for 10 min. The enzymatic hydrolyzate was centrifuged at 9,391 *g* for 10 min at 4°C, and the supernatant was collected as the milk peptide samples and analyzed.

### 2.3 Intact protein analysis

The intact proteins were analyzed based on a recently developed method in our own laboratory. Briefly, an UPLC‐QTOF‐MS system (Waters), including a Waters Acquity H‐class chromatography combined with a Waters Xevo G2 quadrupole time‐of‐flight (QTOF) mass spectrometer, was utilized to analyze the intact milk protein samples. A Waters UPLC BEH300 C4 column (2.1 mm i.d. × 100 mm, 1.7 µm) operated at 80°C was used. The injection volume was 5.0 μl. The elution solvents were 0.1% (v/v) formic acid in deionized water (solvent A) and 0.1% (v/v) formic acid in acetonitrile (solvent B). The gradient program was as: initial condition was 5% B with a flow rate was 0.4 ml/min and kept to 4.9 min; 5 min, 5% B, at 0.2 ml/min; 15 min, 50% B, at 0.2 ml/min; 17 min, 90% B, at 0.2 ml/min; 18 min, 90% B, at 0.3 ml/min; 19 min, 5% B, at 0.4 ml/min; 20 min, 90% B, at 0.4 ml/min; 21 min, 5% B, at 0.4 ml/min; 22 min, 90% B, at 0.4 ml/min; 23 min, 5% B, at 0.4 ml/min; and 25 min, 5% B, at 0.4 ml/min.

The mass spectrometry (MS) analysis was performed in an ESI‐positive ionization mode. The MS operating conditions were as follows: sampling cone voltage, 60 V; capillary voltage, 2.50 kV; extraction cone voltage, 4.0 V; source temperature, 120°C; desolvation temperature, 450°C; and desolvation gas flow, 600 L/hr. The data acquisition range was m/z 50 ~1,500. A MS^E^ scan model was used for obtaining full information of parent and daughter ion using mass spectrometry. A LockSpray™ was performed with leucine enkephalin ([M + H]^+^, m/z 556.2771) solution (200 pg/μl) as the lock mass for mass accuracy.

### 2.4 Hydrolyzed peptide analysis

Peptide samples hydrolyzed from milk protein were analyzed following a previous reported procedure (Dearing et al., 2011). The instruments were similar as that described in the intact protein analysis, except that the column was BEH C18 (2.1 mm i.d. × 100 mm, 1.7 µm) and operated at 30°C, with a run time of 78 min. Mobile phases A was 0.1% (v/v) formic acid in deionized water and mobile phase B was 0.1% (v/v) formic acid in acetonitrile. The injection volume was 5 μl. The gradient program was as follows: 0 min, 2% B, at 0.4 ml/min; 4.9 min, 2% B, at 0.4 ml/min; 5 min, 2% B, at 0.2 ml/min; 65 min, 40% B, at 0.2 ml/min; 67 min, 85% B, flow rate at 0.4 ml/min; 75 min, 100% B, at 0.4 ml/min; 76 min, 2% B, at 0.4 ml/min; and 78 min, 2% B, at 0.4 ml/min. The MS analysis of peptide was performed on same condition of intact protein.

### 2.5 Data processing

The intact protein data obtained from UPLC‐QTOF‐MS were exported by MassLynx version 4.1 software (Waters). The corresponding parameters were as follows: analytical type, combined scan range; peak separation, 0.5 Da; and marker intensity threshold, 10 counts. The peptide data were exported by ProteinLynx Global Server software (PLGS) version 3.0 (Waters). The parameters were as follows: lock mass window, 0.25 Da, low energy threshold, 25 counts, intensity threshold, 75 counts, and peptide tolerance, 10 ppm.

For intact protein samples, a total of 114 chromatographic fingerprints (54 authentic samples and 60 adulterated samples) were obtained from the UPLC‐QTOF‐MS fingerprints. The mass spectrum data of intact protein were exported to obtain a 114 × 2,032 (sample × variables) data matrix. All peptide information was exported to excel file; then, the critical peptide information including peptide sequence and their corresponding intensities were obtained using MATLAB (The MathWorks). The resulting two‐dimensional 114 × 107 peptide matrix (sample × variables) was then applied for statistical analysis. The discrimination of adulterated and authentic samples was evaluated by nontargeted principle component analysis (PCA) method, and the feature information was extracted by targeted pattern recognition PLS‐DA. The peptide and intact protein datasets were merged to perform low‐ and mid‐level data fusion analysis. The spectra were normalized prior to performing chemometrics analyses such as PLS‐DA. In developing the classification models, a total dataset was partitioned into a training set (for calibration) and a test set (for prediction). Each set contained 54 and 60 authentic and adulterated samples, respectively. In order to build accurate PLS‐DA model(s), bootstrapped Latin partitions (BLP) statistic with 10 bootstraps and 5 Latin partitions were to tune optimal latent variable needed for classification (Harrington, 2006). The PCA and PLS‐DA routines were all written in‐house using MATLAB (The MathWorks).

### 2.6 Data fusion

The low‐ and mid‐level data fusions were performed based on the peptide and intact protein data blocks. The procedures of low‐ and mid‐level data fusion are summarized in Figure S1 in the Supporting Information (Borràs et al., 2016). For low‐data fusion, two different data sources were simply concatenated into a single matrix containing information of intact and peptide fingerprints according to a previously reported protocol (Borràs et al., 2016). After that, the meta‐spectra matrix was processed using PCA.

For midlevel data fusion, the variable selection was performed in each data source, and the selected variables were merged to a new data block for model analysis. Variable selection in midlevel data fusion was performed on peptide and intact data blocks using PLS‐DA model based on the normalized datasets. The score and coefficient were acquired from PLS‐DA algorithm, and loading score value was selected to build PCA visualization model (Ramos & Ruisánchez, 2006).

## 3 RESULTS AND DISCUSSION

### 3.1 Principle component analysis of intact protein fingerprints

**Figure 1:**
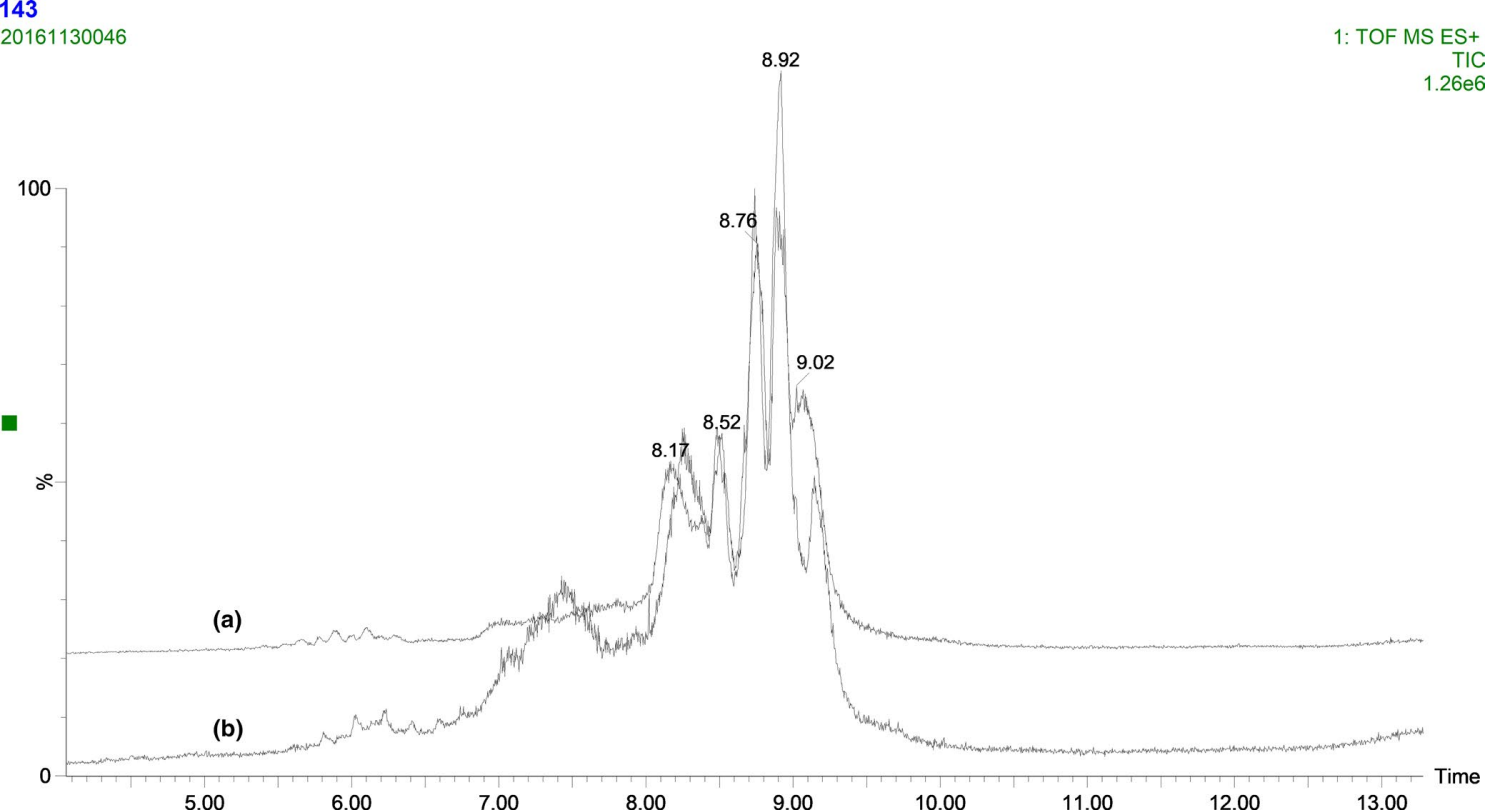
The intact protein total ion chromatogram fingerprints of liquid whole milk (a) and adulterated milk with 10% milk powder (b)

The intact protein constituents of liquid whole milk and its adulterated counterparts were separated and analyzed with UPLC‐QTOF‐MS. The mass chromatograms of representative intact proteins obtained from fresh and 10% adulterated milk samples are shown in Figure 1. The common patterns of UPLC‐MS profiles of liquid whole milk and the corresponding 10% adulterated milk represented that major protein peaks were eluted between 7 and 9.5 min after injection. It appears that the milk adulterated with milk powder could affect the sample composition, since the volatiles and thermal‐sensitive compounds might be changed during the products of milk powders. Several peaks were found in adulterated milk, which revealed that these peaks might be the discriminatory compounds for the liquid whole milk and powder adulterated milk adulterated one. Visually differences were difficult to be discovered and identified based on the chromatograms of liquid whole milk and adulterated milk (Figure 1). In order to differentiate plenty of liquid whole milk with milk powder effectively, the multivariate data analysis was carried out. PCA was used to analyze the data matrix collected from both peptide and intact protein chromatographic fingerprints directly.

**Figure 2:**
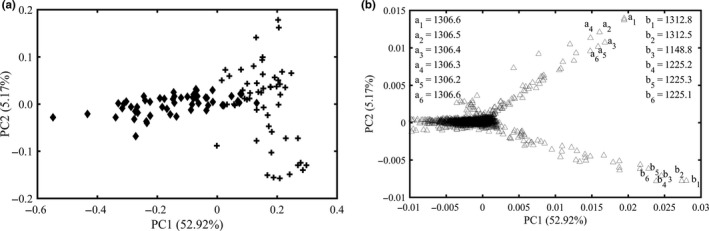
Principle component analysis score plot (a) and loading plot (b) of intact protein fingerprinting of all samples. In the score plot, all the adulterated milk samples were labeled in rhombic, and liquid whole milks were labeled in cross

The PCA score plot of all samples using intact protein fingerprints was shown in Figure 2a. It could be found that the adulterated milk samples were labeled in rhombic and located on the left, and liquid whole milks were labeled in cross and distributed on the right. The first two principal components accounted for 58.09% of the total variance. The contribution rates of the two first components were 52.92% and 5.17%, respectively. The first component played the major role in discriminating fresh and adulterated milk, but there was still a partial overlap between the authentic milk and the adulterated ones.

Loading plot was utilized for further delineating the contribution value for discrimination of adulterated and authentic milk. The loading plot (Figure 2b) of first two components is presented, which highlights that all variables contribute to the first two components. The marked intact protein in the loading plot is important to discriminate adulterated from authentic milk. These intact protein values were m/z 1,306.6, 1,306.5, 1,306.4, 1,306.3, 1,306.2, and 1,306.6 and 1,312.8, 1,312.5, 1,148.8, 1,225.2, 1,225.3, and 1,225.1. These variables are multiple charge peaks of representative milk proteins, and further studies are needed to identify these variables in the intact protein samples.

### 3.2 Principle component analysis of peptide fingerprints

**Figure 3:**
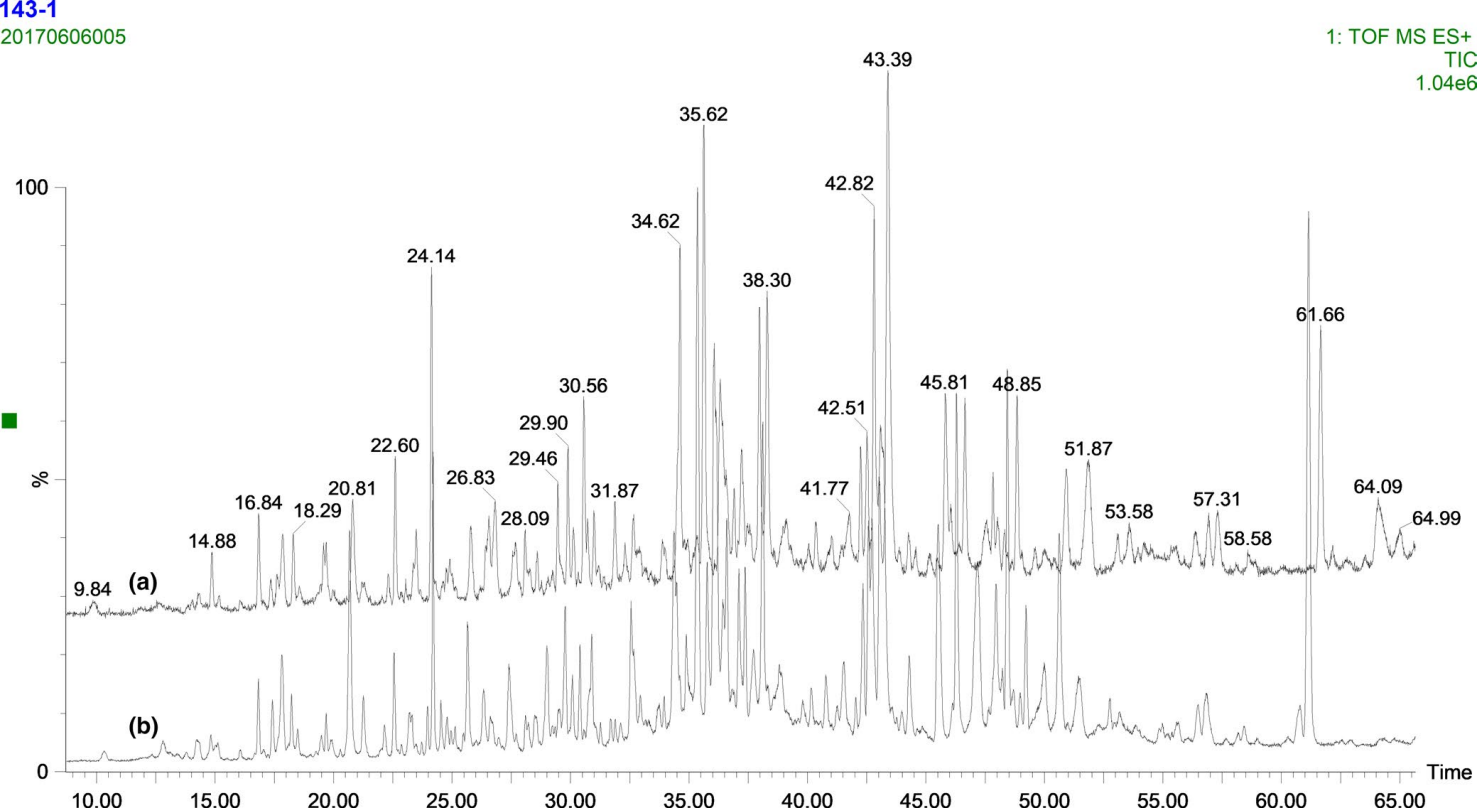
The peptide total ion chromatogram fingerprints of liquid whole milk (a) and adulterated with 10% milk powder (b)

The peptide fingerprints of liquid whole milk and its adulterated counterparts were also obtained by UPLC‐QTOF‐MS (Figure 3). Similar as the results in the intact protein, the mass spectrum differences could not be visually differentiated between the authentic and adulterated samples, even at the high adulteration ratios.

**Figure 4:**
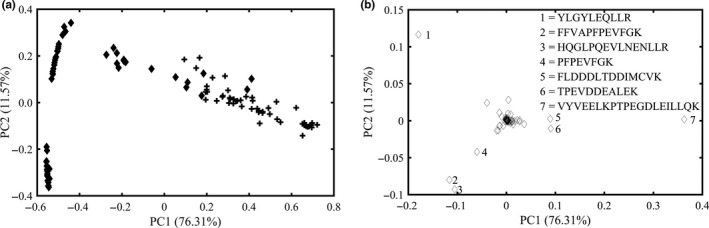
Principle component analysis score plot (a) and loading plot (b) of peptide fingerprinting of all samples. In the score plot, all the adulterated milk samples were labeled in rhombic, and liquid whole milks were labeled in cross

All peptide fingerprints data (114 × 107) were exported for PCA analysis using MATLAB. As shown in Figure 4a, the values of first and second principal components were 76.31% and 11.57%, respectively. Most of the fresh and powdered milk adulterated ones were separated by PC1. The scores plot indicated two different clusters as authentic and adulterated milk were not distinctly separated. A small group of fresh and adulterated milks was still overlapped. Therefore, the pattern recognition method was used to analyze adulterated and authentic milk.

Compared with PCA result using raw peptide fingerprints, intact protein results were slightly poorer. Results from PCA scores represented that the discriminating abilities of fresh and adulterated milk were insufficient by using either independent intact or peptide dataset. These facts suggesting that it was difficult to distinguish adulterated milk from authentic ones from perspective of visualization in the space constructed by the first two largest principal components, possibly due to the similar protein compositions of liquid whole milk and adulterated ones.

Figure 4b shows the corresponding variable loading using peptide fingerprints. The highest loading values of principal score were associated with YLGYLEQLLR, FFVAPFPEVFGK, HQGLPQEVLNENLLR, PFPEVFGK, FLDDDLTDDIMCVK, TPEVDDEALEK, and VYVEELKPTPEGDLEILLQK. The peptide sequence such as FFVAPFPEVFGK, PFPEVFGK, and YLGYLEQLLR presented in α‐s_1_ casein and its inactive modes (proproteins) were tentatively identified. FLDDDLTDDIMCVK also existed in α‐lactalbumin glycosylated polypeptide. The lactosylated site was identified to K112 (Le, Deeth, Bhandari, Alewood, & Holland, 2012). Other peptide sequence including HQGLPQEVLNENLLR, TPEVDDEALEK, and VYVEELKPTPEGDLEILLQK might be assigned to the trypsin by PeptideCutter of ExPASy.

### 3.3 Data fusion

**Figure 5:**
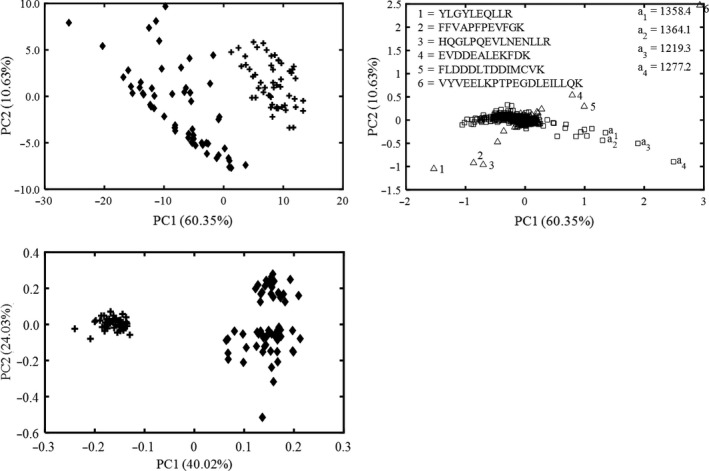
Principle component analysis (PCA) score plot (a) and loading plot (b) of peptide and intact protein fingerprinting of all samples using low‐level data fusion, PCA score plot of peptide and intact protein fingerprinting of all samples using PLS‐DA midlevel data fusion (c), “+,” liquid whole milk, “♦,” adulterated milk

Neither intact protein nor peptide data could achieve a distinct discrimination of adulterated and authentic milk by PCA individually. As the result, the data fusion strategies were used to take advantage of the synergistic effect of the intact protein and peptide information obtained from UPLC‐QTOF‐MS. Firstly, low‐level data fusion was applied to analyze peptide data source combined with intact ones. In low‐level data fusion, the individual data obtained from two different data sources were combined into a new matrix composed of 114 samples and 2,139 variables. The first two principal component values were changed compared with using the individual data source (Figure 5a). The authentic and adulterated milk were grouped in different cluster and the adulterated milk was located on the left side, while liquid whole milk samples were situated on the right side in PCA score plot. Compared with those using the individual observations, the PCA score using the new dataset by low‐level data fusion represented better discrimination result.

Figure 5b represented the representative intact protein ion and peptide sequence from loading plot, which presented the relationship between the loadings of PC1 and PC2. The loading plot from UPLC‐QTOF‐MS spectra acquired with low‐level data fusion has some undefined intact protein peaks at the range of 1,200 ~ 1,400 Da molecular weight. The most important intact proteins for variables PC1 and PC2 were m/z 1,358.4, 1,364.1, 1,219.3, and 1,277.2. These loadings represent the importance of each bin to determine the value the component for the given sample (Ribeiro, Gouveia, Barros, Firmino, & Silva, 2014). For one thing, a part of the loading values are peptide sequence including YLGYLEQLLR, FFVAPFPEVFGK, HQGLPQEVLNENLLR, EVDDEALEKFDK, FLDDDLTDDIMCVK, and VYVEELKPTPEGDLEILLQK. Similar to exploratory PCA loading plot using independent peptide data source, the peptide sequences such as FFVAPFPEVFGK and YLGYLEQLLR presented in α‐s_1_ casein and its precursors. FLDDDLTDDIMCVK also existed in α‐lactalbumin glycosylated polypeptide. The lactosylated site was identified to K112 (Ribeiro et al., 2014). Other peptide sequence including HQGLPQEVLNENLLR and VYVEELKPTPEGDLEILLQK were assigned to the trypsin. It needs to be mentioned that the loading plot for midlevel data fusion results was not presented, since only the information of eigenvalue from the fused peptide and intact protein data were applied to obtain loading value. Thus, the loading values cannot represent any chemical meaning about either the raw intact protein or the peptide of samples, but just the mathematical results fused from PLS‐DA.

In order to increase the discrimination ability between authentic and adulterated milk samples, the midlevel data fusion was carried out by using two different data sources. For midlevel fusion, a variable selection step was independently carried out in different data source. Consequently, the most relevant variables were fused. The detailed procedures were as follows: PLS‐DA model was independently analyzed peptide and intact protein to extract main features. The two datasets were divided into 2/3 training set and 1/3 test set. The optimal latent variables were 4 and 6 for peptide and intact protein data using BLP with 10 bootstraps and 5 Latin partitions. The new matrix was 76 × 20 by merging PLS‐DA score from two different data sources after midlevel data fusion (since only 2/3 of the samples were used as the training set). Compared with dimensionality of raw matrix, the amount of variables significantly decreased. The new data matrix obtained from midlevel data fusion of PLS‐DA score was carried out for PCA analysis. Unlike the previous studies (Márquez et al., 2016), PCA scores were directly used to analyze the midlevel fused feature information, thus resulted in a better understanding of the difference of adulterated and authentic milk in a visual way.

The results were articulated as PCA scores plot in Figure 5c. The first and second components explained 40.02% and 24.03% of total variance. The authentic milk samples have negative scores on the first component, while the adulterated milk samples have positive ones. The results indicated that authentic and adulterated milk could be totally separated by only using the largest component. Compared to the PCA scores using low‐level data fusion, significant difference between the group of adulterated and authentic milk could be observed by using midlevel data fusion. Unlike the low‐level data fusion, the improved performance of the midlevel data fusion showed that combining key information from PLS‐DA model of different data source leading to a better discrimination of liquid whole milk and adulterated ones. These results were confirmed by some previous studies that midlevel data fusion was generally superior compared with the low‐level one (Biancolillo, Bucci, & Marini, 2014; Jin, Rong, & Jiang, 2015). It can be observed that adulterated and authentic milk were distinguished as low as 0.5% adulteration by PCA analysis combined with midlevel data fusion. The data fusion step has to be performed merging the different single dataset into a unique new block of variables. Extracting useful information is most crucial for dealing with midlevel data fusion. The variable selection was needed for midlevel data fusion instead of the all data simply concatenated (Ramos et al., 2008). The application of midlevel data fusion can improve the selection of the best variables. Generally, the discrimination results obtained using a fusion dataset are better than the two separate datasets (Rudnitskaya et al., 2006). This was explained by the fact that some irrelevant data were discarded by midlevel data fusion methodology.

In summary, this study demonstrated that mass spectrometry combined with specific chemometric approaches like data fusion provides sensitive and robust capabilities in differentiating liquid whole milk adulterated with powder adulterated milk. Results represented that only PCA analysis could not discriminate adulterated and authentic milk based on individual intact data or peptide data. By using low‐data fusion from concatenated matrix dataset of peptide and intact fingerprints, adulterated milk could be distinguished from authentic milk effectively. Moreover, compared with low‐level data fusion, the study achieved more satisfactory discrimination between milk and milk adulterated with milk powder based on the midlevel data fusion of peptide and intact protein fingerprints. The advantage of midlevel fusion based on intact and peptide fingerprints combined with PCA analysis has been proved. The combination of peptide and intact protein fingerprints overcame the constraints when applied individually, and the detection limitation could be as low as 0.5% powder adulterated milk adulteration into liquid whole milk. These results represented that data after processed by data fusion have adequate high sensitivity and capable for differentiating very low‐level of powder adulterated milk adulterated to liquid whole milk. Some representative proteins have been detected and tentatively identified as the markers to differentiate milk powder from liquid whole milk. The potential compounds responsible for the discrimination were presumed to be α‐lactalbumin glycosylated polypeptide. The results of this study showed that after processed with data fusion strategy, data collected from the intact protein and peptide fingerprints, analyzed with chemometrics methods, could efficiently differentiate the liquid whole milk adulterated by powder adulterated milk. The utilization of data fusion in processing different complementary data information or many different types of technology is promising for food authentication and worth exploring.

## CONFLICT OF INTEREST

The authors declare that they do not have any conflict of interest.

## ETHICAL APPROVAL

This study does not involve any human or animal testing.
