# Supplementary material for: Detection of milk powder in liquid whole milk using hydrolyzed peptide and intact protein mass spectral fingerprints coupled with data fusion technologies
Source: Food Sci Nutr. 2020 Feb 3;8(3):1471–9. doi: 10.1002/fsn3.1430 (PMC7063352; doi:10.1002/fsn3.1430)
Supplement: Supplementary file 1 [file FSN3-8-1471-s001.docx]

**Detection of presence of milk powder in fresh milk using hydrolyzed peptide and intact protein mass spectral fingerprints coupled with data fusion technologies**

Lijuan Du,^a^ Weiying Lu,^a^ Yaqiong Zhang,^a^* Byan Gao,^a,b^* Liangli (Lucy) Yu^c^

^a^ Institute of Food and Nutraceutical Science, Department of Food Science and Technology, School of Agriculture and Biology, Shanghai Jiao Tong University, Shanghai 200240, China

^b^ China-Canada Joint Lab of Food Nutrition and Health (Beijing), Beijing Technology & Business University (BTBU), Beijing 100048, China

^c^ Department of Nutrition and Food Science, University of Maryland, College Park, MD 20742, United States

***Corresponding author**. Boyan Gao, Yaqiong Zhang

Contact information of the corresponding author: Boyan Gao, Ph.D. Institute of Food and Nutraceutical Science, School of Agriculture & Biology, Shanghai Jiao Tong University, Shanghai 200240, China. Tel: (86) 021-3420-45384041, Email: gaoboyan@sjtu.edu.cn


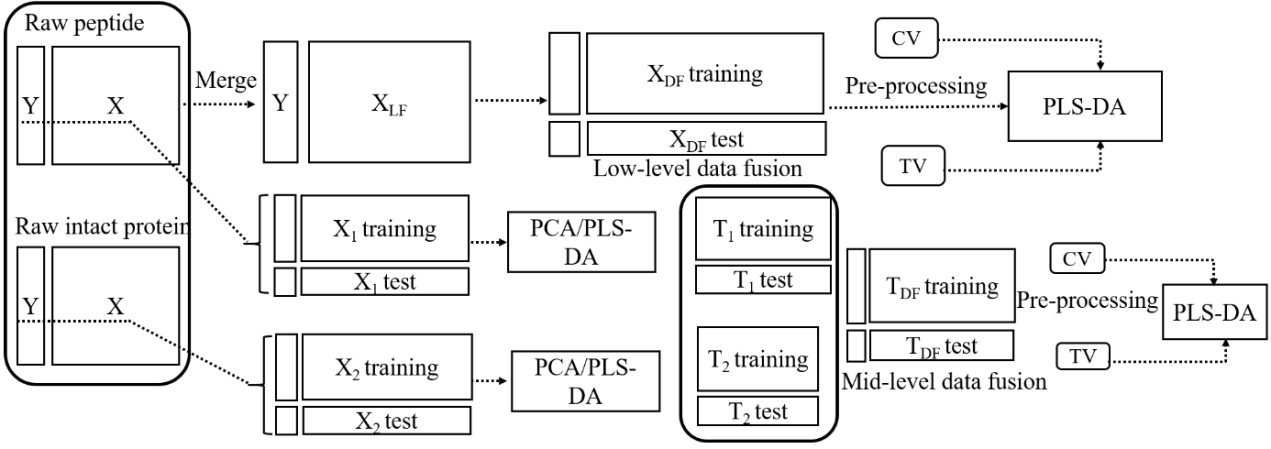


**Figure S1**. The schematic diagram of low-level and mid-level data fusion.
